# Hormonal regulatory networks in spinal cord injury: mechanistic insights, crosstalk, and therapeutic innovations

**DOI:** 10.3389/fendo.2025.1627414

**Published:** 2025-10-17

**Authors:** Wenliang Guo, Yinteng Wu, Shijian Zhao, Jianwen Xu

**Affiliations:** ^1^ Department of Rehabilitation Medicine, The First Affiliated Hospital of Guangxi Medical University, Nanning, Guangxi, China; ^2^ Department of Orthopedic and Trauma Surgery, The First Affiliated Hospital of Guangxi Medical University, Nanning, Guangxi, China; ^3^ Department of Cardiology, The Affiliated Cardiovascular Hospital of Kunming Medical University 6 (Fuwai Yunnan Cardiovascular Hospital), Kunming, Yunnan, China

**Keywords:** spinal cord injury, hormonal signaling, glucocorticoids, neuroinflammation, hormone therapy, bioinformatics

## Abstract

Spinal cord injury (SCI), a debilitating neurological disorder with complex pathophysiology, involves primary mechanical trauma followed by multifactorial cascades of secondary inflammation, oxidative stress, and apoptosis. Hormones have emerged as a research focus in SCI therapeutics due to their neuroprotective properties. As pivotal regulators of cellular signaling, hormones exhibit dual roles in either exacerbating or mitigating secondary damage. This review synthesizes three decades of research, highlighting that hormones such as corticosteroids, melatonin, and estrogen demonstrate significant therapeutic potential in animal models and clinical studies, though controversies persist regarding their efficacy and safety profiles. Key findings include: (1) Glucocorticoids, exemplified by methylprednisolone (MP), suppress inflammation and reduce tissue damage but face skepticism over long-term benefits, with high-dose regimens correlating with significant adverse effects such as gastrointestinal bleeding, hyperglycemia, and metabolic complications; (2) Melatonin exerts multi-target neuroprotection by modulating autophagy, inhibiting apoptosis, and suppressing inflammasome activation; (3) Sex hormones (e.g., testosterone, progesterone) improve functional recovery through metabolic balance regulation and neural regeneration, while estrogen enhances angiogenesis and motor function via the synergistic involvement of multiple receptor-mediated genomic (ERα/ERβ) and non-genomic (GPER) signaling pathways. The non-genomic actions rapidly activate kinase cascades, such as PI3K/Akt-CREB and ERK, which in turn regulate both immediate cellular functions and gene expression profiles, contributing to the overall neuroprotective effects; (4) Combinatorial therapies (e.g., MP with neurotrophic factors) and novel delivery systems (e.g., nanoparticle-based drug carriers) represent promising strategies to optimize therapeutic outcomes. These advances elucidate the multidimensional mechanisms of hormonal interventions while revealing critical challenges, including dose-dependent adverse effects, antagonistic effects in polypharmacy, and unresolved long-term safety concerns. Overall, hormonal therapies for SCI present a “dual-edged sword” of efficacy versus risks, necessitating future innovations in precision regulation and mechanistic exploration to bridge translational gaps.

## 1 Introduction

Spinal cord injury (SCI) initiates with primary mechanical trauma, followed by secondary inflammatory and degenerative cascades involving neuroinflammation, blood-spinal cord barrier (BSCB) disruption, and axonal degeneration. Current treatments, including corticosteroids and immunosuppressants, exhibit limited efficacy and significant adverse effects. Glucocorticoids such as MP were once considered standard acute-phase therapeutics; however, due to an unfavorable risk-benefit profile, they have been downgraded to non-recommended options in major guidelines (1). Hormones, with their pleiotropic roles in inflammation and tissue repair, harbor untapped therapeutic potential. This review systematically examines the roles of hormones in SCI, encompassing molecular mechanisms, clinical evidence, risk-benefit analyses, and cutting-edge technological applications, aiming to inform optimized therapeutic strategies.

## 2 Pathophysiological mechanisms and therapeutic controversies of classical hormones

MP, a representative glucocorticoid, was once regarded as a standard therapy for acute SCI due to its inhibition of lipid peroxidation and inflammatory mediator release. The NASCIS-II protocol recommended a loading dose of 30 mg/kg within 8 hours post-injury, followed by a 24-hour maintenance infusion at 5.4 mg/kg/h, which reportedly improved motor scores (2). However, subsequent studies found no significant motor function improvement in acute traumatic SCI (TSCI) patients under this regimen, alongside markedly increased complication risks (3).

**Figure 1:**
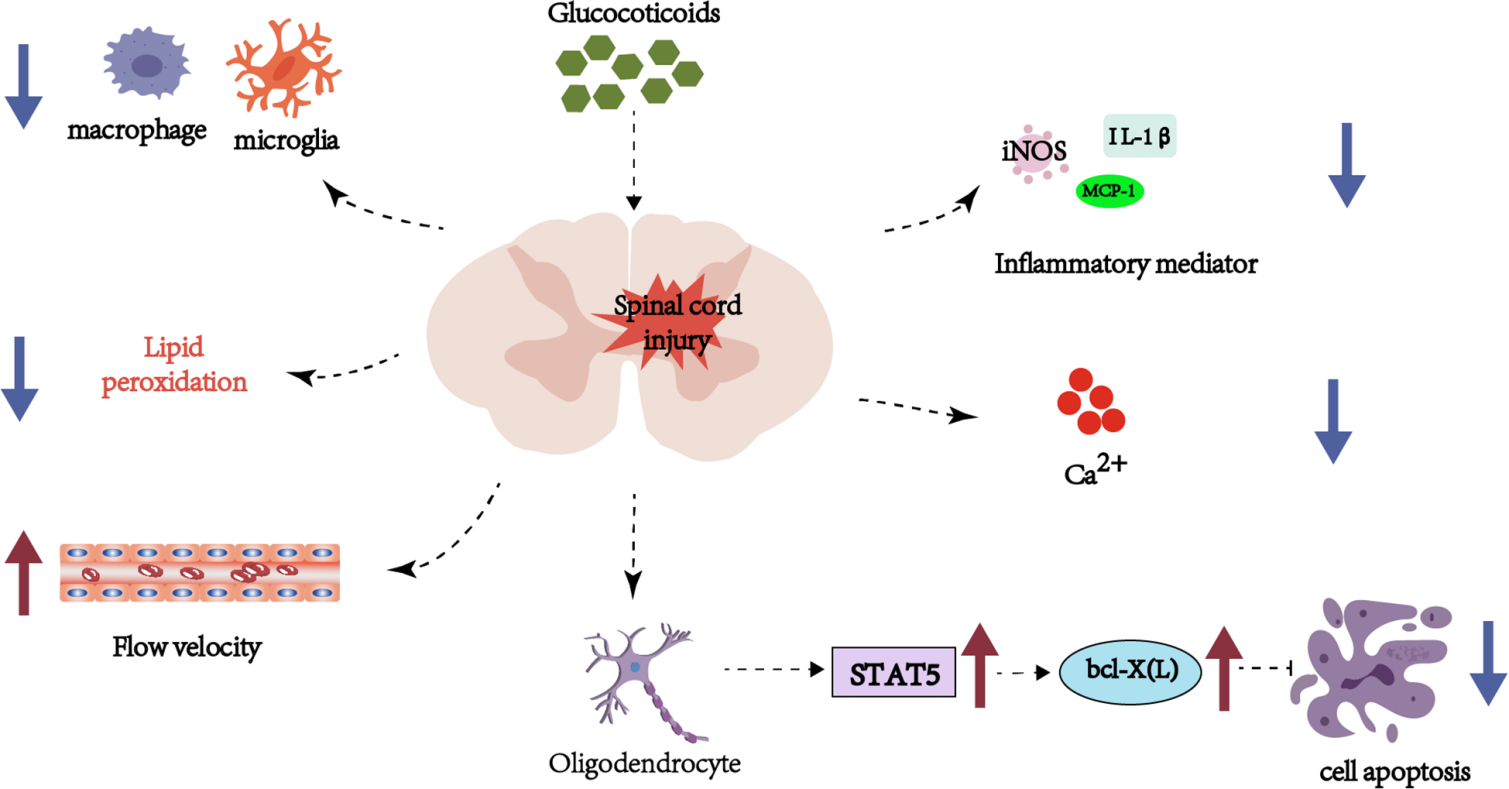
Schematic representation of hormonal and inflammatory pathways in spinal cord injury, emphasizing glucocorticoid involvement, cellular apoptosis, and immune cell interactions.

Multiple studies confirm that MP reduces apoptotic cell death post-SCI (4–6). It lowers levels of malondialdehyde, a lipid peroxidation biomarker (7), and exerts neuroprotection by mitigating axonal damage, enhancing blood flow, reducing calcium influx, and suppressing microglial/macrophage aggregation and inflammatory mediator expression (8). Mechanistically, MP inhibits lipid peroxidation (9) and downregulates pro-inflammatory cytokines (e.g., TNF-α, IL-6) (10), while blocking oxidative stress cascades (11) and suppressing oligodendrocyte apoptosis via the STAT5 pathway (12) (**Figure 1**).

Nevertheless, high-dose MP (>5000 mg), though effective in reducing spinal edema, significantly elevates complication risks (13). In experimental models with a single dose equivalent to that used in humans, the effect found of MP after 24h was an increase in the amount of water due to a decrease in the expression of AQP4, as well as greater damage to the BSCB. Moreover, MP increased the extravasation of plasma components after SCI and enhanced tissue swelling and edema. Notably, MP’s neuroprotection exhibits a critical time window: Two-photon microscopy reveals that administration within 30 minutes post-injury attenuates progressive axonal injury, neuronal death, and microglial/macrophage activation, whereas delayed treatment (>8 hours) drastically diminishes efficacy (14). This narrow timeframe for intervention is further corroborated by time-series transcriptomic analysis, which identifies 8–12 hours post-injury as the optimal window for MP to exert its core immunomodulatory effects (15). These dose- and time-dependent contradictions underscore the necessity for individualized risk-benefit assessments in clinical practice.

Weaver et al. compared the efficacy of MP with anti-CD11d monoclonal antibodies, revealing that while MP preserves spinal cord tissue, it fails to improve neurological function and may antagonize the effects of other immunotherapies (16). Wu et al. demonstrated that 24-hour MP treatment in rats reduced skeletal muscle mass and upregulated atrophy-related genes (FOXO1, MAFbx, MuRF1, and REDD1) (17). High-dose MP significantly elevates risks of infections (e.g., pneumonia), hyperglycemia, and gastrointestinal hemorrhage (18), with higher complication rates observed in pediatric patients (19). Long-term use further correlates with muscle atrophy (17) and diminished ovarian reserve (20). Multicenter studies (3)and clinical guidelines (21) indicate that MP fails to confer significant neurological improvement, challenging its historical status as a “gold standard.” These conflicting outcomes underscore the necessity for rigorous risk-benefit evaluations in MP clinical applications, with future research prioritizing low-dose short-term regimens or combinatorial therapies to mitigate adverse effects.

## 3 Multidimensional regulation of sex hormones: from metabolic modulation to neural regeneration

### 3.1 Testosterone: dual roles in metabolic homeostasis and axonal repair

SCI disrupts hypothalamic-pituitary-gonadal (HPG) axis function, leading to hypogonadism in approximately 60% of male patients (22). Clinical studies demonstrate that testosterone replacement therapy (TRT) significantly increases lean body mass, reduces visceral adiposity, and enhances resting energy expenditure in hypogonadal males with SCI (23, 24). Spinal motor neurons highly express androgen receptor (AR) with predominantly nuclear localization, without significant sexual dimorphism, providing a target for testosterone action (25). Testosterone exerts neuroprotective effects via an AR-dependent mechanism, significantly reducing motor neuron death after injury (26). Clinical studies further confirm that TRT significantly improves motor function in male patients with incomplete SCI (with ASIA Scale scores increasing by 10.9–11.8 points in grades C/D), but shows no efficacy in complete injuries (27), highlighting the reparative potential of AR-mediated testosterone therapy on residual motor pathways. In animal models, testosterone treatment partially reverses MP-induced muscle atrophy and mitigates reductions in muscle fiber cross-sectional area (CSA) (28). Although the precise molecular mechanisms remain unclear, experimental evidence suggests that testosterone regulates the balance between muscle protein synthesis and degradation (29). Furthermore, combining testosterone with resistance training amplifies muscle mass recovery, increasing quadriceps CSA by 9% (30). In neural repair, testosterone administration in adult female rats with spinal contusion preserved dendritic length of motor neurons and maintained muscle mass and fiber CSA, despite no significant effects on lesion volume or motor neuron count (31).

### 3.2 Progesterone: anti-inflammatory pathways and molecular mechanisms of myelin regeneration

Progesterone exerts therapeutic effects in SCI through multifaceted mechanisms. It suppresses pro-inflammatory mediators (e.g., TNF-α, iNOS, COX-2) and glial activation (32, 33), attenuates neuroinflammation via NF-κB pathway inhibition, and achieves neuroprotection by upregulating BDNF expression (34) and preserving neuronal ultrastructure (35). Progesterone promotes multi-mechanistic repair after SCI via classical progesterone receptors. This leads to reduced release of pro-inflammatory cytokines (36). Progesterone enhances remyelination by increasing oligodendrocyte numbers. Treatment groups showed 35% oligodendrocyte presence compared to 7.5% in controls. It also upregulates myelin basic protein expression (37). It supports white matter preservation. Lesion volume is reduced, and white matter sparing increases to 16%, versus 7% in controls. Motor coordination and gait parameters also improve (38). Long-term treatment over 60 days is crucial for functional recovery. It does not induce hyperalgesia. Its clinical safety profile is better than MP. These findings support its translational potential (39). Progesterone also promotes oligodendrocyte differentiation and remyelination (40), alleviates neuropathic pain by inhibiting NMDA receptor hyperactivation (41), and modulates neuropeptide systems (42). Preclinical studies confirm its efficacy in enhancing motor function and axonal survival (43, 44), while a clinical trial combining progesterone with vitamin D demonstrated improved functional recovery in acute SCI (45).

### 3.3 Estrogen: multi-mechanistic neuroprotective effects of estrogen

Among known hormones, estrogens possess some of the most potent neuroprotective effects. Three types of receptors primarily mediate the effects of estrogen: the classical nuclear receptors ERα and ERβ, and the G protein-coupled estrogen receptor (GPER). ERα and ERβ are localized within the nuclei of neurons, particularly in regions associated with reproductive functions such as the dorsal root ganglia, pelvic ganglia, and spinal dorsal horn. Among these, ERα predominates in superficial sensory layers, while ERβ is more abundant in deep autonomic regions, regulating neuropeptide expression and neural plasticity through genomic effects (46). GPER, on the other hand, is an endogenous estrogen receptor located on the endoplasmic reticulum/nuclear membrane. Upon binding with estrogen, it activates nuclear PI3K, leading to the *in situ* synthesis of PIP3. The accumulation of nuclear PIP3 subsequently recruits and activates signaling molecules such as Akt, rapidly regulating transcription factors and gene expression. GPER is an intracellular receptor primarily localized to the endoplasmic reticulum and Golgi apparatus, capable of binding estrogen with high affinity and triggering non-genomic signaling pathways via the Gi/o-EGFR axis to achieve rapid physiological regulation (47). GPER exhibits tissue-specific functions: it promotes proliferation of ER-negative cells in cancer, and plays active roles in cardiovascular protection and metabolic diseases (48). Its ligand promiscuity (e.g., activation by Tamoxifen (TMX)) provides a rationale for developing targeted therapies to circumvent the side effects of traditional estrogen drugs. These receptors together form an integrated nuclear-membrane-intracellular signaling network that precisely regulates the diverse functions of estrogen.

Estrogen exerts neuroprotective effects in SCI through mechanisms including promoting remyelination, anti-apoptosis, pro-angiogenesis, and modulation of microglial and glial cell activity. Estrogen directly promotes remyelination, including inhibiting demyelination processes (49, 50) and enhancing Schwann cell differentiation via the ERβ-ERK1/2 pathway (51). Simultaneously, it reduces oligodendrocyte apoptosis by suppressing the RhoA-JNK3-cJun axis (52), maintaining white matter integrity. Estrogen synergizes with cell therapies by improving the remyelination capacity of stem cells (53) and Schwann cells (54, 55).

Estrogen upregulates the anti-apoptotic protein Bcl-2 via the PI3K/Akt-CREB pathway (56) and inhibits pro-inflammatory cytokines (e.g., TNF-α, IL-1β) and inflammasome activity (57), reducing neuronal apoptosis. Specifically, GPER-mediated non-canonical signaling plays an indispensable role in estrogen’s anti-apoptotic effects, as demonstrated by the findings that the specific GPER agonist G-1 mimics estrogen’s protection against SCI-induced apoptosis while GPER knockdown abolishes this effect, independent of classical nuclear ER pathways (56, 58). In terms of vascular protection, estrogen maintains BSCB integrity by suppressing MMP-9 (59) and stimulates angiogenesis (60). Angiogenesis not only provides nutritional support but also acts synergistically with neural repair processes; for example, the localized nanoparticle-based estrogen delivery platform can reduce glial scar formation and promote axonal regeneration (61), highlighting the interplay of multiple mechanisms.

Estrogen modulates microglial phenotypic polarization through ERα, ERβ, and GPER: suppressing the pro-inflammatory M1 phenotype (reducing expression of CD86, iNOS, and IL-1β) (62) and promoting the anti-inflammatory M2 phenotype (increasing expression of Arg-1 and CD206) (63). The mechanisms involve inhibition of the NF-κB/MAPK pathway (64) and NLRP3 inflammasome activation, as well as delaying the activation of disease-associated microglia through metabolic reprogramming (65). These effects can delay disease progression in experimental autoimmune encephalomyelitis models (66).

Whether the neuroprotective effects of estrogen are sex-specific remains debatable, but most evidence supports the influence of sex differences. For instance, remyelination in female animals does not depend on CXCR4, whereas it depends on the testosterone-CXCR4 axis in males (67, 68); the intrinsic ER expression in microglia is independent of circulating estrogen levels (69), but estrogen loss after menopause exacerbates neuroinflammation (70, 71). Selective estrogen receptor modulators (SERMs) like TMX can mimic the protective effects (72), but supraphysiological doses may exacerbate damage via the ERα/NF-κB pathway (73). These findings provide a basis for developing sex-specific neuroprotective strategies targeting microglia (74). Although some studies suggest limited sex specificity (75), the overall consensus supports considering sex factors in treatment strategies. It is worth noting that the efficacy of TMX is not limited to male animals. Studies by Colón et al. in female rat models demonstrated that TMX administration immediately or 24 hours after SCI similarly improves locomotor recovery and reduces secondary damage (76).

Basic research reveals that 17β-estradiol can significantly reduce immune cell infiltration (e.g., monocytes/macrophages/neutrophils) during the acute phase of SCI, but long-term high-dose use carries carcinogenic risks. TMX can mimic the neuroprotective effects of estradiol in SCI models (e.g., suppressing inflammation, antioxidant, and anti-apoptotic effects) and alleviates tissue damage by downregulating the expression of the Ccr2 and Mmp12 genes in microglia. Ccr2, primarily expressed on microglia and infiltrating macrophages, mediates the recruitment of inflammatory cells to the injury site. In the early phase of SCI (within 24 hours), activated microglia highly express Ccr2, exacerbating neuroinflammation and secondary damage. By downregulating Ccr2, TMX significantly reduces inflammatory cell infiltration, thereby containing the inflammatory response and preserving neuronal and axonal integrity, which aligns with its broad anti-inflammatory action. Mmp12, mainly produced by macrophages/microglia, is involved in extracellular matrix degradation, BSCB disruption, and pro-inflammatory factor activation. In SCI, Mmp12 overexpression aggravates tissue destruction, axonal demyelination, and cell death. By inhibiting Mmp12, TMX helps maintain extracellular matrix stability, reduces tissue damage, and promotes a reparative microenvironment. These mechanisms collectively demonstrate that TMX exerts its neuroprotective effects in the acute phase of SCI (≤24 hours) through multi-targeted interventions in both inflammatory and matrix degradation pathways (76, 77). Administration within 24 hours post-injury can still improve motor function, offering a wider therapeutic window than MP (78). Clinically, although there are no large-scale SCI trials, studies on estrogen use for menopausal syndrome or cardiovascular diseases indirectly support its neuroprotective potential. Animal models show that estrogen treatment improves functional scores, increases axon number and diameter, and enhances motor evoked potentials [with significantly shorter latency (17-fold reduction) and higher amplitude (7-fold increase)]. Future work needs to integrate novel delivery systems (e.g., nano-platforms) and receptor-specific modulation to optimize clinical translation (79).

### 3.4 Gonadotropin-releasing hormone: a potential target for neuroplasticity

GnRH improves SCI outcomes through multi-target mechanisms involving neuroprotection, urological repair, and endocrine regulation. Calderón-Vallejo et al. (80) demonstrated that GnRH enhances motor function in ovariectomized rats by upregulating neurofilament expression and promoting axonal regeneration. Additionally, GnRH exhibits urological protective effects (81), restoring voluntary urination in 68% of SCI rats by reducing bladder wall thickening and renal fibrosis. However, combinatorial therapies may induce complex effects (82). They found that co-administration of GnRH with growth hormone (GH) suppresses motor recovery, underscoring the need for cautious design of multi-hormone regimens. GnRH also modulates the HPG axis: Bauman et al. (83) reported enhanced follicle-stimulating hormone (FSH) responses to GnRH stimulation in SCI males, while Sullivan et al. (50) emphasized that HPG axis central inhibition requires testosterone replacement to improve metabolic health comprehensively.

## 4 Mechanistic roles of metabolic-immune crosstalk hormones

### 4.1 Leptin: bridging metabolic dysregulation and neuroinflammation

Leptin is an energy homeostatic regulatory peptide secreted by white adipocytes. It suppresses appetite and increases energy expenditure by binding to receptors in the hypothalamus. Within the nervous system, the leptin receptor (LepRb) is widely expressed in the hippocampus, cortex, and spinal cord, where it contributes to the regulation of synaptic plasticity (84). Leptin protects neuronal function by inhibiting ATP-induced astrocyte damage, reducing arachidonic acid and prostaglandin E2 release, and activating the JAK2/Stat3 pathway to upregulate caveolin-1 expression (85). Acute leptin treatment decreases caspase-3 activity, suppresses pro-inflammatory molecules, and improves sensory-motor recovery post-SCI (86). Leptin can exacerbate pain by activating microglia (87), whereas blocking leptin signaling suppresses microglial proliferation and alleviates pain. Leptin exhibits a dual role in SCI. Endogenously elevated leptin levels are associated with metabolic syndrome (88), abdominal obesity (89), and neuropathic pain mediated via microglial activation (87). In contrast, acute exogenous leptin administration upregulates caveolin-1 through the JAK2/Stat3 pathway, suppresses ATP-induced inflammatory responses in astrocytes (85), reduces caspase-3 activity and pro-inflammatory cytokine release, promotes oligodendrocyte survival and white matter preservation, and ultimately improves motor functional recovery (86). Its efficacy depends on injury type (protective in complete SCI vs. promotive of pain in root injury) and timing (effective in acute phase) (90). However, long-term leptin treatment may be limited due to exacerbation of lean mass loss (91) or dysregulated bone metabolism (92). Elevated leptin levels in SCI patients correlate with central obesity, metabolic syndrome, and cardiovascular risks (88, 89). This elevation likely stems from sympathetic dysfunction and altered fat distribution, with higher leptin concentrations observed in individuals with more rostral injury levels (93). Notably, leptin positively correlates with lean mass but associates solely with fat mass in sarcopenic obesity, reflecting its metabolic remodeling post-SCI (91). Leptin also enhances bone healing, with increased expression linked to improved callus formation in fracture-SCI models (92).

### 4.2 Melatonin: circadian rhythm-integrated neuroprotection and motor synergy leptin: bridging metabolic dysregulation and neuroinflammation

Melatonin, an indoleamine hormone secreted by the pineal gland, plays essential roles in regulating circadian rhythms—by synchronizing the biological clock via receptors in the suprachiasmatic nucleus to promote sleep—, antioxidant defense through direct scavenging of free radicals (e.g., •OH/ONOO^−^), and activation of enzymes such as SOD and GPx, and immune modulation by suppressing pro-inflammatory cytokines (e.g., TNF-α, IL-6) and enhancing lymphocyte activity. In the context of SCI, these innate functions extend to multi-target neuroprotective mechanisms, including inhibition of inflammasome activation, restoration of mitochondrial dysfunction, and stabilization of the BSCB. Melatonin has emerged as a therapeutic focus in SCI. Its mechanisms include mitochondrial protection, autophagy-apoptosis balance regulation, and inflammasome modulation. Melatonin suppresses NLRP3 inflammasome activity via the Nrf2/ARE pathway, reducing oxidative stress and pro-inflammatory cytokines (e.g., IL-1β, TNF-α) (92), while improving mitochondrial dysfunction and neuronal apoptosis through SIRT1/Drp1 signaling (94). It balances autophagy and apoptosis via PI3K/AKT/mTOR and Wnt/β-catenin pathways, upregulating Beclin-1 and LC3B to enhance cellular clearance (95), and promotes motor neuron survival (96). At the tissue level, melatonin stabilizes the BSCB by inhibiting MMP3/AQP4-mediated microvascular permeability (97) and reduces neuroinflammation via microglial M2 polarization (98).

## 5 Neuroregeneration-oriented hormones and growth factors

### 5.1 Erythropoietin: dual pathways in angiogenesis and anti-apoptosis

EPO, with its anti-apoptotic and anti-inflammatory properties, is a promising therapeutic candidate for SCI. Preclinical studies show EPO inhibits inflammation (reducing myeloperoxidase activity) and apoptosis (lowering caspase-3 activity), while attenuating pathological damage via downregulation of TSP-1 and TGF-β (99). It promotes neural regeneration by upregulating PDGF-β and GFAP expression (64). Clinically, EPO combined with MP improves neurological function and daily living activities in long-term follow-ups (100), with animal studies confirming its superiority over MP monotherapy (101). A clinical trial reported higher primary endpoint achievement in the EPO group versus MP alone (102), and early application (within 6 hours) may enhance efficacy (103), though other trials found no significant differences (104).

### 5.2 Growth hormone: BSCB repair and insulin-like growth factor-1 synergistic mechanisms

GH exerts neuroprotective and reparative effects in SCI through multiple pathways. GH mitigates BSCB disruption and edema formation by reducing tracer extravasation (e.g., Evans blue) and preserving spinal cord evoked potential amplitudes, thus protecting neural conduction (105, 106). Its mechanisms likely involve suppression of vascular hyperpermeability and cellular injury, indicating direct neuroprotective potential in acute phases. GH also indirectly promotes repair by elevating IGF-1 levels, with TiO_2_ nanowire-loaded GH enhancing this effect while reducing BSCB damage and neuronal loss (107). TiO_2_ nanowires are one-dimensional nanomaterials composed of titanium dioxide, with diameters ranging from 1 to 100 nm. They exhibit high specific surface area, good biocompatibility, and photo-responsive properties. Surface functionalization—such as amino modification—confers a positive charge on TiO_2_ nanowires, enabling electrostatic adsorption with negatively charged GH molecules. Alternatively, covalent conjugation strategies like glutaraldehyde cross-linking can be employed to achieve GH immobilization. Preclinical studies suggest GH may improve hemodynamics; for instance, octreotide (a somatostatin analog) increases clitoral and vaginal blood flow post-SCI, though its mechanism differs from GH (108). Notably, SCI patients frequently exhibit GH-IGF-I axis hypoactivity, characterized by blunted GH responses and metabolic abnormalities (e.g., increased adiposity), which GH supplementation may ameliorate (109).

### 5.3 Thyrotropin-releasing hormone: ion homeostasis and neural repair

As a neuropeptide, TRH promotes SCI recovery by regulating monoamine neurotransmitters and ion channel activity. TRH exerts neuroprotective and repair effects through multiple mechanisms in SCI. Basic studies have shown that TRH maintains ion homeostasis, such as reducing cellular edema by enhancing Na^+^-K^+^-ATPase activity (110), and regulates the metabolic balance of TRH and 5-HT in the injured spinal cord to minimize distal neurotransmitter depletion (111). Its anti-inflammatory effects include inhibiting vasogenic edema (112) and downregulating microglial activation to alleviate central pain (113). Additionally, TRH analogs (e.g., CG3703) exhibit therapeutic potential due to their high affinity for spinal TRH receptors, though their efficacy is closely linked to molecular modifications (114). Clinical studies demonstrate that TRH significantly improves motor and sensory functions in patients with incomplete SCI (115). Notably, TRH receptor expression decreases shortly after injury but gradually normalizes with the recovery of endogenous TRH (116), supporting the need for exogenous TRH supplementation. However, the long-term effects of TRH on neuronal excitability remain unclear (117).

## 6 Cross-regulation between the HPA and HPG axes

Following acute SCI, the hypothalamic-pituitary-adrenal (HPA) axis induces excessive glucocorticoid release (e.g., cortisol) via the sympathoadrenal reflex, directly or indirectly inhibiting hypothalamic GnRH secretion and causing central hypogonadism (118). For example, Prüss et al. observed increased cortisol and decreased norepinephrine in mice after acute SCI, suggesting that excessive HPA axis activation disrupts HPG axis function through neuroendocrine mechanisms (118). HPG axis dysfunction exacerbates metabolic disorders: HPG axis inhibition (e.g., low testosterone) is closely associated with increased body fat and metabolic syndrome in SCI patients, further promoting sustained HPA axis activation. Sullivan et al. found that low testosterone in young male SCI patients is primarily due to hypothalamic-pituitary drive deficiency and correlates significantly with increased body fat (50). Bauman et al.confirmed that the pituitary gland in SCI patients shows enhanced FSH response but insufficient luteinizing hormone response to GnRH, indicating that hypothalamic regulation impairment may be related to HPA axis negative feedback (49). Through GnRH dose-response experiments, Bauman et al. identified specific differences in pituitary responses among SCI patients, highlighting the need for optimized interventions (e.g., glucocorticoid antagonism or testosterone supplementation) to break the vicious cycle (119).

In summary, interactions between the HPA and HPG axes after SCI form a closed-loop of “increased glucocorticoids-gonadal inhibition-metabolic abnormalities,” necessitating combined targeted therapies to improve outcomes.

## 7 Combination therapies and novel delivery strategies: overcoming therapeutic efficacy limitations

### 7.1 Combination therapies

The limitations of single-agent hormone therapies have driven researchers to explore combination regimens and innovative delivery technologies. Combination therapies can overcome the efficacy ceiling of monotherapy. MP, a classic anti-inflammatory agent, exhibits marked variability in its combinatorial effects. For instance, Gorio et al. demonstrated that MP antagonizes the neuroprotective effects of EPO by suppressing EPO receptor upregulation or interfering with its anti-apoptotic pathways (120). In contrast, a clinical study by Xiong et al. revealed that co-administration of EPO and MP significantly improved neurological function and daily living capacity in ischemia-reperfusion injury (100). This paradox suggests that MP’s immunosuppressive properties may exert dual-phase effects depending on pathological stages—suppressing inflammation acutely while potentially impeding regenerative signaling pathways.

### 7.2 Combination strategies targeting antioxidant and immunomodulatory pathways show greater synergistic potential

Carnosine is an endogenous dipeptide (β-alanyl-L-histidine) widely present in muscular and neural tissues, known for its antioxidant, anti-glycation (inhibiting AGEs formation), and pH-buffering capacities. Irisin is an exercise-induced myokine released through the cleavage of FNDC5 protein, primarily involved in regulating energy metabolism (promoting browning of white adipose tissue), neuroprotection (upregulating BDNF), and anti-inflammatory pathways (suppressing NF-κB). In the context of SCI, the combination of MP and carnosine alleviates neural damage by elevating irisin levels, likely through synergistically enhancing antioxidant defenses and promoting the secretion of neurotrophic factors. This pathway has been further confirmed by Albayrak et al (121). Teixeira et al. further demonstrated that MP combined with granulocyte colony-stimulating factor (G-CSF) significantly enhanced motor function and reduced inflammatory cell infiltration, indicating complementary mechanisms between G-CSF’s neuroregenerative effects and MP’s anti-inflammatory actions (122). Similarly, Genovese et al. confirmed that melatonin combined with dexamethasone synergistically suppressed neutrophil infiltration and apoptosis, mitigating secondary injury (123). In summary, successful combination therapies require meticulous optimization of temporal coordination, target complementarity, and dosage regimens.

## 8 Clinical translation potential of targeted delivery systems

To circumvent systemic side effects of conventional drug administration, MP-loaded nanoparticles (e.g., PLGA-MP) and *in situ* gels (e.g., fibrin/chitosan composites) have emerged as research priorities. Cox et al. utilized estrogen-loaded nanoparticle patches targeting injury sites, reducing glial scar formation and promoting axonal regeneration while avoiding systemic toxicity from high-dose estrogen (74). Similarly, Karabey-Akyurek et al. developed an MP-nano-fibrin gel for localized delivery, achieving efficacy comparable to high-dose systemic MP with significantly reduced adverse effects (124). Qin et al. designed Nano-MP, a prodrug-based system selectively targeting injured areas, which not only enhanced neuroprotection in rat models but also avoided glucocorticoid-induced muscle atrophy and osteoporosis (125). Wang et al. reported carrier-free nanoparticle MP(2)-TK@RU NPs integrated with the antioxidant rutin, enabling ROS-responsive MP release to simultaneously suppress inflammation, oxidative damage, and promote functional recovery (126). Chvatal et al. achieved deep MP penetration using PLGA nanoparticles combined with hydrogels (127), while Zhai et al. developed a microneedle-CD-MOF system to breach the dura barrier for precise controlled release (128). Despite diverse strategies, these studies collectively demonstrate that targeted delivery systems can overcome the limitations of conventional therapies. Future efforts should prioritize validating long-term safety and clinical applicability to advance nanodelivery technologies from experimental to clinical stages.

## 9 Conclusion and prospect

Hormonal therapies exhibit multi-layered mechanisms in SCI repair, ranging from anti-inflammatory actions to neuroregeneration. However, their clinical application remains challenged by inconsistent efficacy and significant side effects, necessitating precise strategies to harness their therapeutic potential. Research on hormonal interventions for SCI has evolved from single-molecule approaches to complex regulatory networks. Beyond classical glucocorticoids, it is now well described that sex hormones like estrogen or its SERMs, like TMX, as well as other hormone (e.g., melatonin, GH, and leptin) contribute to neuroprotection and repair via multi-target mechanisms. Future studies should focus on: 1. Elucidating hormone-cytokine-epigenetic crosstalk to develop multi-pathway synergistic agents; 2. Leveraging nanotechnology and gene editing to overcome delivery barriers and advance novel delivery platforms; 3. Deciphering receptor-specific signaling pathways for targeted agonists/antagonists using OMICs and bioinformatics approaches; 4. Optimizing combination strategies, such as integrating hormones with stem cells or biomaterials.
